# An analysis of Hall mobility in as-grown and annealed n- and p-type modulation-doped GaInNAs/GaAs quantum wells

**DOI:** 10.1186/1556-276X-7-529

**Published:** 2012-09-25

**Authors:** Fahrettin Sarcan, Omer Donmez, Mustafa Gunes, Ayse Erol, Mehmet Cetin Arikan, Janne Puustinen, Mircea Guina

**Affiliations:** 1 Department of Physics, Science Faculty, Istanbul University, Vezneciler, Istanbul, 34134, Turkey; 2 Optoelectronics Research Centre, Tampere University of Technology, Korkeakoulunkatu, Tampere, 33720, Finland

**Keywords:** GaInNAs, Electronic transport, Thermal annealing, Modulation-doped quantum wells, 72.10-d, 72.20.Fr, 71.55.Eq.

## Abstract

In this study, we investigate the effect of annealing and nitrogen amount on electronic transport properties in n- and p-type-doped Ga_0.68_In_0.32_N_*y*_As_1 − *y*_/GaAs quantum well (QW) structures with *y* = 0%, 0.9%, 1.2%, 1.7%. The samples are thermal annealed at 700°C for 60 and 600 s, and Hall effect measurements have been performed between 10 and 300 K. Drastic decrease is observed in the electron mobility of n-type N-containing samples due to the possible N-induced scattering mechanisms and increasing effect mass of the alloy. The temperature dependence of electron mobility has an almost temperature insensitive characteristic, whereas for p-type samples hole mobility is decreased drastically at *T* > 120 K. As N concentration is increased, the hole mobility also increased as a reason of decreasing lattice mismatch. Screening effect of N-related alloy scattering over phonon scattering in n-type samples may be the reason of the temperature-insensitive electron mobility. At low temperature regime, hole mobility is higher than electron mobility by a factor of 3 to 4. However, at high temperatures (*T* > 120 K), the mobility of p-type samples is restricted by the scattering of the optical phonons. Because the valance band discontinuity is smaller compared to the conduction band, thermionic transport of holes from QW to the barrier material, GaAs, also contributes to the mobility at high temperatures that results in a decrease in mobility. The hole mobility results of as-grown samples do not show a systematic behavior, while annealed samples do, depending on N concentration. Thermal annealing does not show a significant improvement of electron mobility.

## Background

The dilute III-N-As alloys have been widely investigated due to the unusual fundamental physical properties [1,2]. It has been proven that the material properties are suitable for many device applications such as laser sources emitting at 1.3 to 1.55 μm, detector, and optical amplifiers for fiber-optic communication systems [3-5]. Moreover, Ga_1 − *x*_In_*x*_N_*y*_As_1 − *y*_ with an energy band gap of approximately 1 eV has become of particular importance for future use in lattice-matched GaInP/GaAs GaInNAs/Ge 4-junction tandem solar cells [6].

Although N behaves as an isovalent impurity in Ga(In)As host lattice, its atomic size and electronegativity differ from that of the As atoms. Therefore, N acts as deep center localized above extended conduction band states of the host semiconductor. The interaction between localized N level and delocalized conduction band states restructures the conduction band of the host semiconductor, splitting the conduction band into two sub-bands, *E*_*−*_ and *E*_*+*_. *E*_−_ band constitutes the fundamental band edge of Ga(In)NAs alloy. Only 1% of N causes approximately 150-meV band shrinkage, therefore gives a great flexibility to tailor the band gap of the host material. *E*_−_ band has a highly non-parabolic energy dispersion relation. The non-parabolicity is responsible for the enhanced electron effective mass in dilute nitrides.

On the other hand, the presence of nitrogen atoms in Ga(In)As lattice makes it difficult to obtain high quality materials due to dissimilarities in atomic radius and electronegativities between N and As atoms of the host semiconductor. Therefore, the optical and electrical properties are strongly affected by the presence of the N atoms. The incorporation of N into the structure leads to form defects such as single N and N-N pairs, N-As, and N-As_Ga_ due to low growth temperature and dissimilarity between N and As. In the multilayer structures, strain between adjacent constitute layers is another source of defects. Incorporation of N into Ga_1 − *x*_In_*x*_As reduces the strain of Ga_1 − *x*_In_*x*_As layer grown on GaAs [7]. Even though the addition of N strongly affects the electron effective mass, the presence of N has a negligible effect on the valance band and hole effective mass according to the *k·p* model [8-13]. An effective method is post or *in situ* thermal annealing which improves optical and crystal quality [14-16].

In this study, we experimentally investigated the effects of N amount and thermal annealing on carrier mobility of n- and p-type modulation-doped Ga_0.68_In_0.32_N_*y*_As_1 − *y*_/GaAs (*y* = 0%, 0.9%, 1.2%, 1.7%) using Hall effect measurements. A drastic effect of N on electron mobility is observed and attributed to the enhanced electron effective mass which is supported by the experimental findings from Shubnikov-de Haas measurements. The presence of N also causes a slight decrease in the hole mobility. Because it is thought that N has a negligible effect on the valance band, observed reduction of mobility is ascribed to the alloy scattering and interface scattering due to the presence of strain between GaAs and Ga_0.68_In_0.32_N_*y*_As_1 − *y*_. Temperature dependence of electron mobility is almost temperature insensitive, whereas hole mobility follows the trend in 2D hole gas of InGaAs. At low temperature, hole mobility is found to be much higher than electron mobility. A significant improvement for low temperature hole mobility is obtained as a result of optimum thermal annealing conditions. As for electrons, thermal annealing increased the mobility at the interest of temperature range. Our results exhibited that thermal annealing is an effective way to enhance carrier mobility. To the best of our knowledge, we observed the highest mobility in dilute nitrides.

## Methods

**Figure 1:**
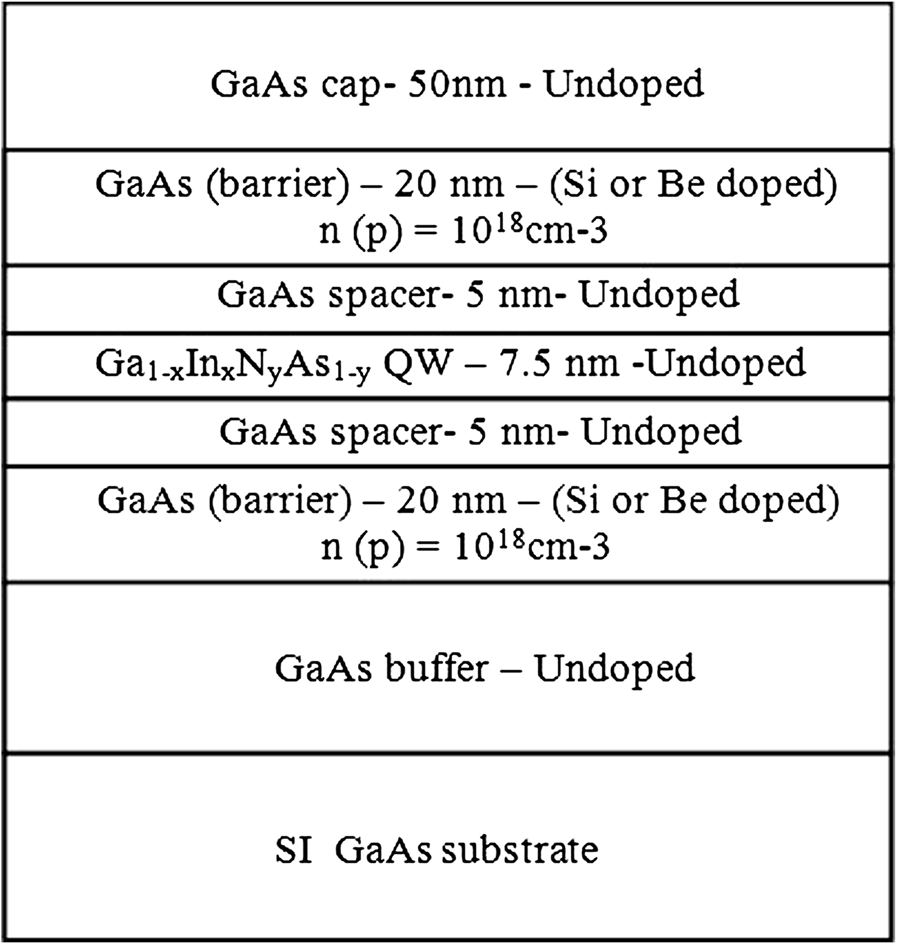
The layer structures of the samples.

All samples investigated in this study are listed in Table 1. The samples were grown on semi-insulating GaAs (100) substrates using solid source MBE equipped with a radio frequency plasma source for nitrogen incorporation. The structures comprised of 7.5-nm thick quantum well (QW) with indium concentration of 32% and a varying nitrogen concentration (N% = 0, 0.9, 1.2, 1.7) and 20-nm doped (Be for p-type and Si for n-type) GaAs barriers. The doped GaAs barriers are separated from the QW by a 5-nm undoped spacer layer, as shown in Figure 1, to reduce the effect of ionized impurity scattering. Growth temperatures for GaAs, Ga_1-x_In_x_As and Ga_0.68_In_0.32_NyAs1 − y layers were 580°C, 540°C and 475°C, respectively. Rapid thermal annealing was done at 700°C for 60 and 600 s.

**Table 1:** Samples used in the investigations listed along with the corresponding sample codes

**Description**	**Type**	**As-grown**	**Annealed**	**Annealed**
			**(60 s at 700°C)**	**(600 s at 700°C)**
Ga_0.68_In_0.32_As	p	TPR	TPRA	TPRB
Ga_0.68_In_0.32_N_0.009_As_0.991_		TP09	TP09A	TP09B
Ga_0.68_In_0.32_N_0.012_As_0.988_		TP12	TP12A	TP12B
Ga_0.68_In_0.32_N_0.017_As_0.983_		TP17	TP17A	TP17B
Ga_0.68_In_0.32_As	n	TNR	TNRA	TNRB
Ga_0.68_In_0.32_N_0.009_As_0.991_		TN09	TN09A	TN09B
Ga_0.68_In_0.32_N_0.012_As_0.988_		TN12	TN12A	TN12B
Ga_0.68_In_0.32_N_0.017_As_0.983_		TN17	TN17A	TN17B

TPR, p-type N-free sample; TNR, n-type N-free sample.

Samples were fabricated in the form of a Hall bar with lengths of 1.75 mm and width of 0.2 mm. Ohmic contacts were formed by alloying Au/Zn and Au/Ge/Ni for p- and n-type materials, respectively. Hall effect measurements were performed in the temperature range between 10 and 300 K to study the low field transport properties of the samples. The current flowing through the sample was kept relatively low (*I* < 100 μA) to ensure ohmic conditions. Both the mobility and carrier concentration were found to be independent of the current and magnetic field at all temperatures. A steady magnetic field was applied perpendicular to the plane of the samples.

## Results and discussions

**Figure 2:**
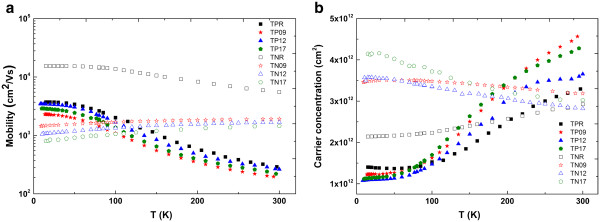
**Temperature dependence.** (**a**) Carrier mobility and (**b**) carrier concentration of as-grown p- and n-type samples.

Figure 2a shows the hole and electron mobility of the as-grown samples with various nitrogen concentrations as a function of temperature. Because the two-dimensional electron gas (2DEG) is undoped and ionized donors are spatially separated from 2DEG, electron mobility is higher than that in bulk Ga_1 − *x*_In_*x*_N_*y*_As_1 − *y*_ semiconductor. The hole mobility exhibits the characteristic temperature dependence of the 2D carriers as observed in N-free GaInAs heterostructures and incorporation of N decreases the hole mobility. In fact, it is thought that N does not have a significant effect on valance band; therefore, effective mass of holes is not affected by the presence of N. However, we observed a decrease in hole mobility for all N-containing p-type samples compared to the N-free p-type sample. If we consider that N only has a negligible effect on the hole effective mass, the origin of this trend can be related to interface scattering and N-induced alloy scattering in the sample. Hall mobility of the p-type N-free sample (TPR) is lower than that in the n-type N-free sample (TNR), because of the larger hole effective mass compared to the electron effective mass. On the other hand, N-containing p-type samples have higher mobility at low temperature range than corresponding n-type semiconductor. In N-containing structures, conduction band is affected from the presence of the N atoms and, as a result, the structure has larger electron effective mass than the N-free structures. We have found the effective masses for TNR, TN09, and TN12 from the Shubnikov-de Haas measurements as 0.042, 0.055, 0.067 m, respectively. The fact that the effective mass enhances with increasing N concentration is a dominant mechanism for decreasing mobility in n-type dilute nitrides, along with enhanced N-related alloy scattering and interface scattering. Therefore, lower electron mobility than the corresponding hole mobility at low temperatures can be attributed to mainly enhanced electron effective mass.

All p-type samples have a very weak temperature dependence of mobility below *T* = 30 K then decreases rapidly with increasing temperature, as expected for the enhanced polar optical phonon scattering. At high temperatures (*T* > 250°C), the hole mobility of as-grown samples takes approximately the same value. Because the valance band offset is so small in GaInNAs/GaAs QW, as the temperature increases, holes can escape from the quantum well via thermionic emission. Therefore, observed high temperature mobility is a result of parallel conducting channels due to the full ionization of acceptors further away from the depletion regions as well as the thermionic emission of holes from the quantum wells over the shallow barriers [12]. On the contrary, electron mobility has a much weaker temperature dependence. This behavior may be associated with the high electron concentration-induced screening effects. Sun et al. showed that electron mobility in GaInNAs/GaAs QW is mainly determined by the N-induced alloy and interface roughness scattering at low temperatures and limited by polar optical phonon and alloy scattering at high temperatures, analyzing analytically the temperature dependence of mobility [12]. Temperature dependence of 2D carrier concentration of as-grown samples is shown in Figure 2b. The observed temperature dependence of hole concentration tends to be constant from 10 to 70 K. However, at high temperatures (*T* > 70 K), carrier density increases. The reason for this behavior might be associated with the increasing concentration of ionized acceptor (Be-doped), generating free holes. The lowest carrier concentration at intermediate temperature range has been observed for N-free sample grown at optimized temperature of InGaAs. In the p-modulation-doped sample at high temperatures, the hole concentration represents a combination of the 2D hole gas in the quantum well and the holes in the wide GaAs barriers due to the full ionization of acceptors further away from the depletion regions as well as the thermionic emission of holes from the quantum wells over the shallow barriers. On the other hand, GaAs barrier is deeper for GaInAs/GaAs QW. Thus, for N-free sample, contribution of holes in the barrier layer will be less.

At low temperatures (*T* < 40 K), electron concentration of n-type samples is almost constant because of frozen impurity atoms (Si), then it increases as temperature increases for N-free sample, but decreases for N-containing samples. Enhancement of electron concentration with increasing N concentration is due to the flattening of conduction band, giving rise to the enhanced density of state of electron at conduction band edge. However, it is difficult to make a comparison of temperature dependence of N-containing samples because they do not exhibit a systematic behavior and tend to slightly decrease with increasing temperature in contrast to the reference sample. As seen from Figure 2a, there is not a systematic trend of hole mobility with increasing N concentration in contrast to the n-type samples. It is another indication that mobility is dominated by other effects. In order to understand this trend, we take into account the alloy scattering and strain effects in p-type samples. Alloy scattering potential for ternary (N-free samples) is given by the following equation:

(1) $$U_{A_{1-x}B_{x}C}=\frac{b}{4\pi \varepsilon_{0}}\left[\frac{Z_{\text{A}}}{r_{\text{A}}}-\frac{Z_{\text{B}}}{r_{\text{B}}}\right]e^{-k_{S}R}\text{,}$$

where *Z*_A_ and *Z*_B_ are valence numbers, *r*_A_ and *r*_B_ are covalent radii, *k*_s_ is the Thomas-Fermi screening wave number. The factor *b* = 1.5 accounts for the fact that the Thomas-Fermi approximation overestimates screening for small interatomic distances [17]. The Thomas-Fermi wave number is given by Equation 2:

(2) $$k_{s}^{2}=\frac{1}{4}{\left(\frac{\pi }{3}\right)}^{\frac{1}{3}}\frac{a_{\text{B}}}{n_{0}}\text{,}$$

where *n*_0_ is the valence electron density, and *a*_B_ is the Bohr radius. The valence electron density is then given by the equation below:

(3) $$n_{0}=\frac{32}{a_{0}^{3}}\text{,}$$

where *a*_0_ is the lattice constant. Lattice constant and *R* are obtained applying Vegard's law as

(4) $$a_{0}=\left(1-x\right)a_{\text{A}}+xa_{\text{B}}$$

(5) $$R=\frac{1}{2}\left[r_{\text{C}}+xr_{\text{B}}+\left(1-x\right)r_{\text{A}}\right]\text{,}$$

where *r*_C_ is the covalent radii of the third atom in the alloy.

Considering that effect of N is negligible in p-type samples, conventional alloy potential for ternary alloys can be adapted to quaternary alloy using the equation below:

(6) $$\begin{array}{c} {\left|U_{A_{1-x}B_{x}C_{y}D_{1-y}}\right|}^{2}=x(1-x)y^{2}{\left|U_{\mathrm{ABC}}\right|}^{2}+x(1-x){\left(1-y\right)}^{2}{\left|U_{\mathrm{ABD}}\right|}^{2}+x^{2}y(1-y){\left|U_{\mathrm{BCD}}\right|}^{2}\\ +{\left(1-x\right)}^{2}y(1-y){\left|U_{\mathrm{ACD}}\right|}^{2}\text{.} \end{array}$$

**Figure 3:**
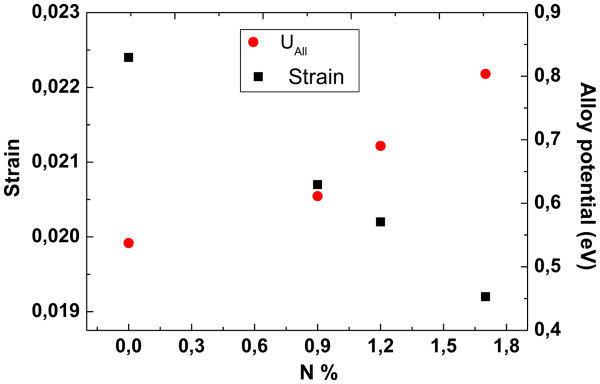
Strain and alloy potential versus N%.

According to Equation 6, alloy potential increases with N concentration, but hole mobility does not decrease monotonically (see Figure 2a). Therefore, in order to interpret the N dependence of hole mobility, we also considered the strain between GaAs and GaInNAs layers. In our structures, addition of N decreases strain; therefore, interface roughness scattering decreases as seen in Figure 3[18]. 

Figure 3 shows that there is an opposite contribution of two effects with increasing N amount. An interplay between two mechanisms affects the hole mobility. Even when alloy potential is the lowest, strain takes the largest value for TP09. In this case, hole mobility suffers from interface roughness. The highest mobility is observed for TP12. For this sample, both mechanism affect moderately, and the reduction in strain enhances the hole mobility. As for TP17, strain takes the lowest value, but alloy scattering drastically deviates from the value in N-free sample, therefore suppresses the improvement due to lower strain. As a result, the value of hole mobility is determined from an inter-relation between these two effects and their contribution changes with N unequally.

**Figure 4:**
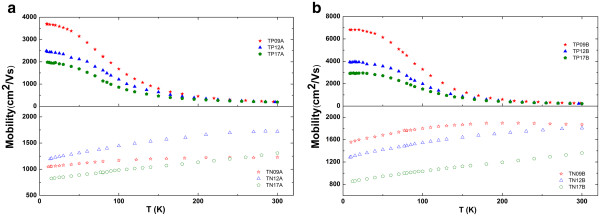
**Temperature dependence of p- and n-type annealed samples.** Annealing time is (**a**) 60 s and (**b**) 600 s.

Thermal annealing has been carried out to improve optical and crystal quality as a standard technique. As far as we know, there is no other research in the literature regarding thermal annealing on electronic transport properties of Ga_1 − *x*_In_*x*_N_*y*_As_1 − *y*_ alloys. It is well-known that thermal annealing causes to re-arrange neighbor atoms in N environment and re-shape the QW from square to parabolic as a result of Ga-In interdiffusion, leading to a blueshift. Kurtz et al. claimed that during growth, N is initially placed into the lattice sites surrounded by gallium atoms, forming N-Ga bond that causes lattice mismatched, i.e., strain between layers. On the other hand, during annealing, In-N bond becomes favorable [16]. TEM results revealed that the interface roughness increases with N, but thermal annealing makes interface smoother [19,20]. Figure 4 shows the result of Hall mobility of n- and p-type annealed samples. Two different annealing duration times used were 60 and 600 s. As seen from Figure 4, the best improvement has been observed for TP09, and the low temperature hole mobility value is the highest in the literature. This result is expected because hole mobility of as-grown TP09 is the most affected from alloy scattering and high-strain induced interface roughness scattering. As for other p-type samples, a significant improvement has been observed for only 600-s process; thermal annealing for 60 s deteriorates the low temperature hole mobility for both TP12A and TP17B. During the thermal annealing, there may be several competing mechanism, and therefore, interpretation of mobility changes under thermal annealing is not straight forward. The fact that re-arrangement of N environment after thermal annealing decreases strain is not the only mechanism, Ga-In inter-diffusion causes a change in the strain as well. Because the diffusion rate of Ga and In atoms is different and depend on the carrier concentration, the direction of change in strain is not obvious. Also the re-arrangement of N environment changes the strength of the interaction between localized N level and the conduction band states. Therefore, it is thought that thermal annealing for 60 s is not the optimum temperature. On the other hand, the best improvement has been obtained for 600 s. After 600-s annealing is applied, hole mobility decreased with N amount. Sun et al. analyzed the temperature dependence of electron mobility results and found that the interface scattering was especially dominant at low temperatures [12]. Since we observed an improvement at low temperature hole mobility, we can speculate that strain is reduced for all samples after the 600-s annealing process, and hole mobility is affected by N-induced alloy scattering. 

As for n-type samples, thermal annealing does not have a drastic effect on the electron mobility. This is a strong indication that dominant effect on electron mobility is due to the enhanced electron mass. Again, the highest electron mobility is obtained after the 600-s annealing process was applied. The contribution of both interface scattering and alloy scattering may be decreased as a result of annealing; therefore, electron mobility is slightly enhanced over all temperature range. Under the light of our discussion, it can be concluded that the proper annealing time for all the samples is 600 s.

## Conclusions

In conclusion, we have investigated temperature dependent Hall mobility for as-grown and annealed p- and n-type modulation doped Ga_0.68_In_0.32_As/GaAs and Ga_0.68_In_0.32_N_*y*_As_1 − *y*_/GaAs QW structures containing different N concentrations. The investigated samples were annealed at 700°C for 60 and 600 s, respectively. The Hall measurement results showed that the presence of N affects both electron and hole mobility. At low temperature range, hole mobility is much higher than the corresponding electron mobility. Hole mobility follows the temperature dependence of 2D hole gas in InGaAs. At high temperatures, hole mobility does not show any dependence of N amount and take its lowest value which is an indication of transport that takes place in GaAs barrier layer. Results also indicated that alloy scattering and interface scattering are dominant mechanisms that explain the behavior of temperature dependence of both as-grown and annealed samples. As for n-type sample, low mobility is a result of enhanced effective mass. The fact that thermal annealing only enables a slight increase on electron mobility is an indication that alloy scattering and interface roughness are not the main mechanisms to limit the mobility. The best improvement on carrier mobility is obtained for 600-s annealing time.

## Abbreviations

2DEG: two-dimensional electron gas; QW: quantum well.

## Competing interests

The authors declare that they have no competing interests.

## Authors’ contributions

FS and OD carried out the experiments and contributed to the writing of the article. MG (Istanbul University) fabricated the samples. AE designed the structure of the samples, conducted the experimental work, and wrote the most part of the article. MCA supervised the experimental work. JP and MG (Tampere University of Technology) grew and annealed the samples. All authors read and approved the final manuscript.
